# Premenstrual symptoms and associated factors in female students in Saudi Arabia: a cross-sectional study

**DOI:** 10.3389/fpubh.2025.1699948

**Published:** 2025-12-08

**Authors:** Vanitha Innocent Rani, Akshaya Saikannan, Abeer S. Aseeri, Eman Baleegh Meawad Elsayed, Gehan EL Nabawy Ahmed Moawad, Rasha Gamal Ahmed Abdelgawwad, Amutha Chellathurai, Kamaldeen Nasrin Nisha, Mona M. Abd El-Maksoud, Safaa Ibrahem Shattla, Francis Moses Rajappa

**Affiliations:** 1 Department of Community and Psychiatric Nursing, Nursing College, King Khalid University, Muhayil Asir, Saudi Arabia; 2 Department of Anatomy, Vinayaka Mission’s Medical College and Hospital, Vinayaka Mission Research Foundation (DU), Karaikal, Puducherry, India; 3 Department of Adult Care and Advanced Nursing, Nursing College, King Khalid University, Muhayil Asir, Saudi Arabia; 4 Faculty of Nursing, Gerontological Nursing, Mansoura University, Mansoura, Egypt; 5 Department of Maternity and Child Nursing, Nursing College, King Khalid University, Muhayil Asir, Saudi Arabia; 6 Faculty of Nursing, Maternal and Newborn Health Nursing, Menofyia University, Shibin el Kom, Egypt; 7 Department of Maternity and Child Nursing, Nursing College, King Khalid University, Abha, Saudi Arabia; 8 Saveetha College of Nursing, Saveetha University, Chennai, India; 9 College of Applied Medical Sciences, King Khalid University, Muhayil, Saudi Arabia; 10 Department of Community Health Nursing, Nursing College, King Khalid University, Abha, Saudi Arabia; 11 Faculty of Nursing, Helwan University, Helwan, Egypt; 12 Department of Community and Psychiatric Nursing, College of Nursing, Khamis Mushait, Saudi Arabia; 13 Faculty of Nursing, Menoufia University, Shibin el Kom, Egypt; 14 St. Xavier College of Nursing, Kumbakonam, Tamilnadu, India

**Keywords:** premenstrual syndrome, female students, nursing, prevalence, symptoms, Saudi Arabia, university students

## Abstract

**Background:**

Premenstrual syndrome (PMS) is a common condition affecting the physical, psychological, and behavioral health of reproductive-aged women. This study aimed to assess the association between sociodemographic and menstrual characteristics and the severity of PMS symptoms among female students.

**Methods:**

A cross-sectional study was conducted among 370 female college students. Data were collected through a structured questionnaire covering sociodemographic factors, menstrual characteristics, and PMS symptoms across three domains: physiological, psychological, and behavioral. Descriptive statistics, ANOVA, and multiple linear regression analyses were performed to identify associated factors.

**Results:**

Departments (94.6%). The majority had a normal BMI (65.4%), and 76.5% reported moderate menstrual flow. ANOVA results indicated significant associations between PMS symptoms and BMI, marital status, menstrual cycle length, and menstrual flow. In the regression models, overweight or obese students demonstrated significantly lower physiological (*β* = −0.283, *p* = 0.031), psychological (*β* = −0.348, *p* = 0.018), and behavioral symptom scores (*β* = −0.481, *p* < 0.001) compared with underweight peers. Students with heavy menstrual flow reported higher symptom scores across all domains (physiological: *β* = 0.931, p < 0.001). Overweight or obese students exhibited lower symptom scores compared to their underweight peers (*β* = −0.283, *p* = 0.031). Additionally, students from non-healthcare departments demonstrated significantly higher psychological symptoms (*β* = −0.744, *p* = 0.001), and those who experienced early menarche (≤13 years) showed more behavioral symptoms (*β* = 0.424, *p* = 0.013) than healthcare students.

**Conclusion:**

PMS symptoms among female students are significantly influenced by BMI, menstrual flow characteristics, department of study, and age at menarche. These findings highlight the importance of targeted health education and support programs that consider these factors to effectively manage PMS symptoms in this population.

## 1 Introduction

Premenstrual syndrome (PMS) is a multifaceted condition that affects a significant number of menstruating women, presenting a broad spectrum of symptoms across physiological, cognitive, affective and behavioral domains. It is characterized by the recurrence of the symptoms in the luteal phase of the menstrual cycle, typically resolving upon or shortly after the onset of menstruation. Globally, it is estimated that between 30 and 90% of women experience at least one PMS symptom during their reproductive years, with moderate to severe PMS affecting approximately 20 to 40% of them.

PMS was first recognized in the 1930s, with initial research focusing primarily on its physiological manifestations, such as bloating, headaches and breast tenderness (1–3). Over the years, the scope of research has expanded to include psychological and behavioral symptoms, such as mood swings, anxiety, depression, irritability and difficulties in concentration (4, 5). A study emphasized the significant impact of PMS on cognitive functioning, particularly during the luteal phase, where women experience heightened psychological reactivity and diminished cognitive performance (6).

PMS poses a public health challenge due to its impact on women’s quality of life, productivity, and social functioning. A meta-analysis highlighted a particularly high prevalence in Asian and Middle Eastern countries, including Iran, India, and Saudi Arabia (7). Furthermore, a systematic review found that PMS contributes significantly to school absenteeism, decreased academic performance, and workplace inefficiency, especially among young women and university students (8, 9).

In university students, PMS is of particular concern due to its potential interference with academic responsibilities and social interactions. Female students are at a critical period where academic achievements and social engagements are pivotal for future success (10, 11). The stress of balancing academic demands with personal and social responsibilities, coupled with PMS symptoms, can exacerbate the psychological and physiological burden (12). Various studies have reported high prevalence rates of PMS among university students, indicating that PMS had significantly lower quality of life in all domains (7, 8, 13).

The psychological and behavioral symptoms of PMS, in particular, have received increased attention in recent years, as they can be more debilitating than the physiological symptoms. Research demonstrated that women with PMS are more likely to experience mood disturbances, including irritability, anxiety, and depression, which can lead to significant impairment in social and academic functioning (11). PMS affects a significant portion of menstruating women, particularly those in high-stress environments such as universities.

Menstrual-related depression is recognized as a significant concern for female university students. Research has shown that a considerable proportion of this population is at elevated risk for mental health issues associated with the menstrual cycle (14). The World Health Organization also acknowledges that many female students globally experience menstrual-related psychological disturbances (15, 16). In Saudi Arabia, several studies have reported a high prevalence of PMS among university students, with notable impacts on psychological, behavioral, and physiological well-being (13, 16–20) While most studies emphasize that PMS negatively impacts students’ overall quality of life, they often overlook its contribution to impairments in home responsibilities and academic productivity. Collectively, these findings emphasize the need for targeted interventions and support systems for managing PMS among female students in the Middle Eastern context.

In Saudi Arabia, the cultural context and societal expectations regarding women’s health add another layer of complexity to understanding the experience of PMS. While the topic of menstruation is often considered taboo, there has been a growing interest in reproductive health issues among young Saudi women (13). Previous studies have found that women with PMS are less likely to seek medical advice or discuss their symptoms due to cultural stigmatization and a lack of awareness (21).

Given the significant impact of PMS on health, daily activities and academic performance among female university students, this study aims to investigate the prevalence, severity and impact of PMS among female undergraduate students at King Khalid University, Saudi Arabia. his study aimed to assess the prevalence and severity of PMS among students, examine the correlations among physiological, psychological, and behavioral symptoms, and explore the associations between PMS severity and demographic characteristics.

## 2 Materials and methods

### 2.1 Study design, participants and sampling

A descriptive cross-sectional study was conducted to assess the prevalence, severity, and impact of PMS among female undergraduate students at the King Khalid University, Saudi Arabia. A total of 370 female undergraduate students were recruited using a convenience sampling technique, ensuring proportional representation across all academic year levels. The recruitment was carried out in classroom settings within the university, allowing for ease of access and high response rates.

Eligible participants were Saudi nationals, aged 18 to 25 years, and enrolled full-time in the university’s undergraduate program. Inclusion criteria required participants to: (1) have experienced a menstrual period during the last two consecutive months; (2) be able to read and understand English or Arabic; and (3) provide informed consent to participate. Exclusion criteria included: (1) current pregnancy; (2) a self-reported history of chronic medical conditions such as diabetes, hypertension, heart disease, or psychiatric disorders including depression or anxiety; and (3) current use of hormonal therapy. Additionally, individuals who declined consent or were uncooperative during data collection were excluded from the study.

Ethical approval was obtained from the Institutional Review Board (IRB) of King Khalid University (ECM#2024-2,403). The study was conducted in accordance with the ethical principles of autonomy, confidentiality, and informed consent. There was no anticipated physiological or behavioral risk to participants. However, there were minimal anticipated psychological risks to participants. Those who experienced distress were offered access to university counseling services.

### 2.2 Measures

Data were collected using the Premenstrual Syndrome Scale (PMSS), a standardized, self-administered questionnaire comprised of 40 questions with three sub-scales namely, physiological, psychological and behavioral symptoms was used to assess the presence of PMS. Responses were recorded using a five-point Likert scale: “never” (1), “rarely” (2), “sometimes” (3), “very often” (4), and “always” (5). A total score of 80 or above was used to indicate the presence of premenstrual syndrome (PMS), and a mean item score of 2.1 or higher was considered indicative of elevated symptom severity in this study. The PMSS scale is publicly accessible tools available online. The tool also included questions on demographic, gynecologic, and obstetric pro-files, including age, height, weight, marital status, class year, place of residence, age at menarche, average menstrual cycle length, number of bleeding days, and menstrual flow type. The PMSS has demonstrated strong psychometric properties, including high test–retest reliability, temporal stability, and internal consistency in prior research (22). In the present study, the scale ex-habited excellent internal reliability, with Cronbach’s alpha of 0.89.

Self-administered questionnaires were employed to assess the research variables. Demographic and menstrual health-related variables used in the study were categorized to facilitate subgroup analysis. Age was grouped into three categories: below 20 years, 20–23 years, and 24–26 years. Marital status was classified into “Single” and “Married and Others.” Academic Grade level was divided from Grade 1 to Grade 5 based on the year of study. Body Mass Index (BMI) was calculated using the formula weight (kg) divided by height (m^2^) and categorized into three groups: Underweight (BMI < 18.5), Normal (18.5–24.9), and Overweight/Obese (BMI ≥ 25), following WHO standards. Age at menarche was grouped into ≤13 years, 14–15 years, and ≥16 years. The number of bleeding days during menstruation was categorized as 3 days, 4–5 days, and over 6 days. Menstrual flow was also classified based on participant perception into Mild, Moderate, and Heavy.

### 2.3 Data collection

Data collection was conducted from October to November 2023, and completing the survey required approximately 15–20 min. The questionnaire was distributed electronically using Google Forms. Participants were encouraged to ask questions and seek clarification where needed. They were given adequate time to review the consent form and decide whether to participate without pressure to rush their decision. Participation in the study was entirely voluntary. Written informed consent was obtained from all participants after a clear explanation of the study’s objectives, procedures and potential benefits. The participants were informed that the data collected would be kept strictly confidential, with no identifying information such as names or addresses included to maintain anonymity. They were assured that no physiological, psychological, or psychological harm would result from their participation, and they had the right to refuse or withdraw at any point without consequence.

### 2.4 Data analysis

Data was analyzed using the Statistical Package for Social Sciences (SPSS) version 26. Descriptive statistics, including frequencies and percentages, were used to summarize participants’ socio-demographic characteristics and the prevalence of premenstrual symptoms. To examine relationships between PMS symptom severity (physiological, psychological, and behavioral) and selected demographic variables (age, weight, height, marital status, grade), Chi-square (χ^2^) tests were conducted. Additionally, Pearson correlation analysis was used to assess the strength and direction of associations between the different PMS symptom domains. A *p*-value < 0.05 was considered statistically significant.

## 3 Results

### 3.1 General demographic characteristics of the female students

Table 1 presents the demographic and menstrual characteristics of the study participants (*N* = 370). The majority were between the ages of 20–23 years (58.1%), followed by those aged >20 years (39.5%), with only a small proportion aged 24–26 (2.4%). Regarding BMI, most participants had a normal BMI (65.4%), while 20.5% were overweight or obese, and 14.1% were underweight. Most participants were single (95.7%), with only 4.3% married or classified under others. Academic year distribution showed the highest representation from third-year students (50.8%), followed by first-year (23.0%), fourth year (19.5%), fifth year (4.1%), and second-year students (2.7%). In terms of menarche age, over half (54.9%) had their first menstruation at or before 13 years, 34.9% between 14–15 years, and 10.3% at 16 years or later. The average menstrual cycle length was ≤27 days for 43.8%, 28 days for 38.6%, and ≥29 days for 17.6%. The majority reported moderate menstrual flow (76.5%), while 13.5% experienced mild flow and 10.0% had heavy flow.

**Table 1:** Distribution of socio-demographic and menstrual characteristics of female students (*n* = 370).

Characteristics		Frequency/M	Percent/SD
Age	>20	146	39.5
	20–23	215	58.1
	24–26	9	2.4
Marital status	Single	354	95.7
	Married and others	16	4.3
Grade	1	85	23.0
	2	10	2.7
	3	188	50.8
	4	72	19.5
	5	15	4.1
BMI	Underweight	52	14.1
	Normal	242	65.4
	Overweight/obesity	76	20.5
Age of menarche	≤13	203	54.9
	14–15	129	34.9
	≥16	38	10.3
Number of days bleeding	3 days	162	43.8
	4 to 5 days	143	38.6
	Over 6 days	65	17.6
Menstrual flow	Mild	50	13.5
	Moderate	283	76.5
	Heavy	37	10.0
Symptoms (M, SD)	Physiological	2.71	0.90
	Psychological	2.65	0.99
	Behavior	2.25	0.97

The mean scores for premenstrual symptom dimensions were as follows: physiological symptoms (*M* = 2.71, SD = 0.90), psychological symptoms (*M* = 2.65, SD = 0.99), and behavioral symptoms (*M* = 2.25, SD = 0.97).

### 3.2 Characteristics of menstrual cycle and symptoms

Table 2 presents a comparative analysis of physiological symptoms related to menstruation across various participant demographics and menstrual characteristics. Significant differences in physiological symptoms were observed based on marital status, body mass index (BMI), number of days of bleeding, and menstrual flow. Unmarried participants exhibited higher mean scores (Mean = 2.923) for physiological symptoms compared to their married counterparts (Mean = 2.680), with a statistically significant *p*-value of 0.003. Regarding BMI, underweight individuals reported the highest symptom severity (Mean = 3.229). These results were statistically significant (*p* < 0.001). Other factors such as age group, grade, and age at menarche showed no statistically significant association with physiological symptoms.

**Table 2:** Comparison of physiological symptoms on menstruation period and general characteristics of the participants and cycle characteristics.

Characteristics		Physiological			
		Mean	SD	t/F	*p*
Age (Years)	>20	2.637	0.891	2.094	0.125
	20–23	0.891	2.737		
	24–26	2.737	0.881		
Mari	Single	2.923	0.822	8.867	0.003
	Married and others	2.680	0.889		
Grade	1	3.356	0.849	1.720	0.145
	2	2.607	0.835		
	3	3.263	0.908		
	4	2.675	0.935		
	5	2.844	0.825		
BMI	Underweight	3.229	1.246	4.077	0.018
	Normal	2.836	0.980		
	Overweight/obesity	2.615	0.890		
Age of menarche	≤13	2.513	0.801	1.622	0.199
	14–15	2.774	0.933		
	≥16	2.595	0.800		
Number of days bleeding	≤27	2.752	0.995	6.648	0.001
	28	2.520	0.942		
	≥29	2.868	0.834		
Menstrual flow	Mild	2.834	0.839	14.458	<0.001
	Moderate	2.349	0.988		
	Heavy	2.690	0.844		

Table 3 presents the comparison of psychological symptoms experienced during menstruation across various demographic and menstrual cycle characteristics. Among all variables, BMI, and menstrual flow showed statistically significant associations with psychological symptoms (*p* < 0.05). BMI showed the highest mean symptom score among underweight students (*M* = 3.148), followed by normal BMI (*M* = 2.865), and overweight/obese students (*M* = 2.550). Menstrual flow was also significantly associated with psychological symptoms (*p* < 0.001), with participants reporting mild flow showing the highest symptoms (*M* = 2.719), followed by heavy flow (*M* = 2.611), and moderate flow (*M* = 2.432). Differences across academic year, bleeding days, and menarche age were not statistically significant.

**Table 3:** Comparison of psychological symptoms on menstruation period and general characteristics of the participants and cycle characteristics.

Characteristics		Psychological			
		Mean	SD	t/F	*p*
Age (Years)	>20	2.645	0.970	1.163	0.314
	20–23	0.970	2.638		
	24–26	2.638	0.990		
Mari	Single	2.834	0.934	1.948	0.164
	Married and others	2.638	0.985		
Grade	1	2.990	1.013	1.732	0.142
	2	2.667	0.961		
	3	3.258	0.681		
	4	2.558	1.031		
	5	2.778	0.896		
BMI	Underweight	3.148	1.200	3.852	0.022
	Normal	2.865	1.111		
	Overweight/obesity	2.550	0.964		
Age of menarche	≤13	2.021	0.938	2.250	0.107
	14–15	2.692	1.002		
	≥16	2.525	0.948		
Number of days bleeding	≤27	2.879	1.008	1.599	0.204
	28	2.549	1.029		
	≥29	2.740	0.954		
Menstrual flow	Mild	2.719	0.946	9.181	<0.001
	Moderate	2.432	1.155		
	Heavy	2.611	0.920		

Table 4 presents a comparison of behavioral symptoms experienced during menstruation in relation to participants’ general and menstrual characteristics. Among age group, >20 years reported higher behavioral symptom scores (*M* = 2.292) than others and Single participants (*M* = 2.461) also had greater symptom severity than married or others. Underweight students exhibited the highest behavioral scores (*M* = 3.130) compared with normal and overweight/obese peers. Early menarche (≤13 years) was linked to higher behavioral symptoms (*M* = 2.879), and those with mild menstrual flow (*M* = 2.341) reported greater symptoms. All of the variables were shown significant (*p* < 0.05) except grade (*p* = 0.856) than those with moderate or heavy flow. Grade level and duration of bleeding showed no significant differences.

**Table 4:** Comparison of behavioral symptoms on menstruation period and general characteristics of the participants and cycle characteristics.

Characteristics		Behavioral			
		Mean	SD	t/F	*p*
Age	>20	2.292	0.983	4.520	0.012
	20–23	0.983	2.180		
	24–26	2.180	0.920		
Marital status	Single	2.461	1.008	4.773	0.030
	Married and Others	2.224	0.949		
Grade	1	2.760	1.214	0.333	0.856
	2	2.276	0.968		
	3	2.417	0.756		
	4	2.203	0.999		
	5	2.267	0.890		
BMI	Underweight	3.130	1.364	7.270	<0.001
	Normal	2.564	1.065		
	Overweight/obesity	2.112	0.905		
Age of menarche	≤13	2.879	1.002	5.709	0.004
	14–15	2.265	0.973		
	≥16	2.091	0.873		
Number of days bleeding	≤27	2.682	1.104	1.111	0.330
	28	2.164	1.014		
	≥29	2.298	0.914		
Menstrual flow	Mild	2.341	0.954	5.206	0.006
	Moderate	2.063	0.946		
	Heavy	2.220	0.921		

### 3.3 Correlation between the menstrual symptoms

Table 5 displays the correlation coefficients between behavioral, psychological, and physiological menstrual symptoms among female students. The results indicate statistically significant positive correlations across all three symptom categories at *p* < 0.001. Behavioral symptoms showed a strong correlation with psychological symptoms (*r* = 0.753) and a moderate correlation with physiological symptoms (*r* = 0.608). Additionally, psychological symptoms were highly correlated with physiological symptoms (*r* = 0.780).

**Table 5:** Correlation between the menstrual symptoms of the female students.

Symptoms	Physiological	Psychological	Behavioral
Physiological	1	0.753**	0.608**
Psychological		1	0.780**
Behavioral			1

**p < 0.001.

### 3.4 Association between the menstrual symptoms and covariates

**Figure 1:**
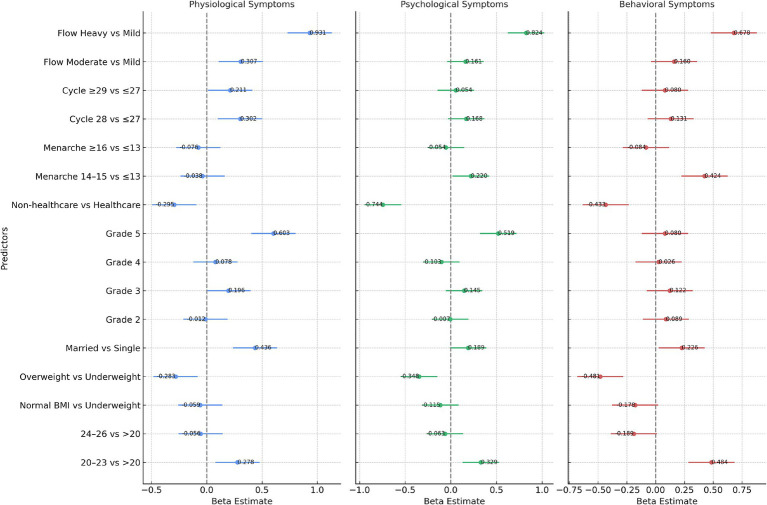
Forest plot for association of premenstrual symptoms and outcomes.

Table 6 presents the multiple linear regression analysis revealed several significant associations between socio-demographic factors and menstrual symptoms among female students (Figure 1). For physiological symptoms, students classified as overweight/obese reported significantly lower symptom scores (*β* = −0.2829, *p* = 0.031). Similarly, those in Grade 5 showed higher physiological symptom scores (*β* = 0.6032, *p* = 0.039). The length of the menstrual cycle of 28 days (*β* = 0.3021, *p* = 0.002) and heavy menstrual flow (*β* = 0.9305, *p* < 0.001) were also significantly associated with increased physiological symptom severity. For psychological symptoms, overweight/obese students (*β* = −0.3475, *p* = 0.018), and those from non-healthcare departments (*β* = −0.7442, *p* = 0.001) had significantly lower scores. However, students with heavy menstrual flow (*β* = 0.8235, *p* < 0.001) showed significantly higher psychological symptom scores.

**Table 6:** Association between the level of physiological symptoms and selected socio demographic variables of female students.

Variables	Physiological				Psychological				Behavioral			
	Estimate	SE	*t*	*p*	Estimate	SE	*t*	*p*	Estimate	SE	*t*	*p*
Interceptᵃ	2.385	0.185	12.924	<0.001	2.691	0.206	13.054	<0.001	2.398	0.202	11.883	<0.001
Age												
>20	1.000				1.000				1.000			
20–23	0.278	0.329	0.844	0.399	0.329	0.367	0.896	0.371	0.484	0.360	1.347	0.179
24–26	−0.056	0.121	−0.464	0.043	−0.063	0.135	−0.467	0.641	−0.189	0.132	−1.435	0.152
BMI												
Underweight	1.000				1.000				1.000			
Normal	−0.059	0.154	−0.383	0.702	−0.115	0.172	−0.670	0.503	−0.178	0.169	−1.053	0.293
Overweight/obesity	−0.283	0.131	−2.166	0.031	−0.348	0.146	−2.382	0.018	−0.481	0.143	−3.369	<0.001
Marital status												
Single	1.000				1.000				1.000			
Married & Others	0.436	0.243	1.793	0.074	0.189	0.272	0.695	0.487	0.226	0.266	0.851	0.396
Grade												
1	1.000				1.000				1.000			
2	−0.012	0.265	−0.043	0.965	−0.007	0.296	−0.024	0.981	0.089	0.290	0.306	0.760
3	0.196	0.170	1.153	0.250	0.145	0.190	0.765	0.445	0.122	0.186	0.657	0.512
4	0.078	0.138	0.568	0.571	−0.103	0.154	−0.669	0.504	0.026	0.150	0.173	0.863
5	0.603	0.291	2.076	0.039	0.519	0.325	1.599	0.111	0.080	0.318	0.251	0.802
Department												
Healthcare	1.000				1.000				1.000			
Non-healthcare	−0.295	0.204	−1.449	0.148	−0.744	0.227	−3.272	0.001	−0.433	0.223	−1.944	0.053
Age of menarche												
≤13	1.000				1.000				1.000			
14–15	−0.038	0.155	−0.244	0.808	0.220	0.174	1.270	0.205	0.424	0.170	2.497	0.013
≥16	−0.076	0.098	−0.777	0.037	−0.054	0.109	−0.496	0.020	−0.084	0.107	−0.782	0.435
Length of one menstruation cycle												
≤27	1.000				1.000				1.000			
28	0.302	0.099	3.047	0.002	0.168	0.111	1.515	0.131	0.131	0.108	1.205	0.229
≥29	0.211	0.127	1.657	0.098	0.054	0.142	0.380	0.704	0.080	0.139	0.577	0.564
Menstrual flow type												
Mild	1.000				1.000				1.000			
Moderate	0.307	0.134	2.289	0.023	0.161	0.150	1.073	0.284	0.160	0.147	1.093	0.275
Heavy	0.931	0.190	4.907	<0.001	0.824	0.212	3.886	<0.001	0.678	0.207	3.267	0.001

Reference categories: Age (>20), BMI (Underweight), Marital status (Single), Grade (1st), Department (Healthcare), Age of menarche (≤13), Menstrual cycle length (≤27), and Menstrual flow (Mild).

Regarding behavioral symptoms, overweight/obese students (*β* = −0.4811, *p* < 0.001) and those from non-healthcare departments (*β* = −0.4326, *p* = 0.053) were associated with significantly lower behavioral symptom scores. Early menarche at age 14–15 was positively associated with behavioral symptoms (*β* = 0.4242, *p* = 0.013). Additionally, heavy menstrual flow had a significant positive association with behavioral symptom severity (*β* = 0.6775, *p* = 0.001).

## 4 Discussion

This study investigated the prevalence and severity of premenstrual syndrome (PMS) among female university students, focusing on physiological, psychological, and behavioral symptoms, their intercorrelations, and associations with demographic variables. Consistent with previous regional and international studies, our findings revealed a high prevalence of PMS, with 87% of participants experiencing symptoms higher than the 35.6% reported in a Saudi-based study (16, 18, 23).

Various studies have reported high PMS prevalence among university students, indicating that PMS significantly lowers quality of life across all domains (9–11, 24, 25). The most frequently reported physiological symptoms in our study fatigue, abdominal cramps, and food cravings are consistent with findings from studies in Iran and Pakistan, which also observed that physiological symptoms are the most dominant in PMS (15, 16, 24). A Saudi study similarly noted fatigue and pain as common complaints among university-aged women (14). Moreover, the present study found strong positive correlations between physiological, psychological, and behavioral symptom domains, suggesting that although PMS interventions should be multipronged, addressing physiological symptoms may also reduce psychological and behavioral symptoms (2). These findings reinforce the interconnected nature of PMS symptomatology and emphasize the importance of integrated, culturally sensitive management approaches in young female populations.

Our findings reported the psychological symptoms, including mood swings, crying spells, and sleep disturbances, were also highly prevalent. These findings are supported by previous research indicating high rates of psychological instability and irritability during the premenstrual phase (19). Another study found a significant association between psychological symptoms and academic stress in Iranian students (6, 20) in Saudi Arabia emphasized the psychological burden PMS places on young women, leading to social and academic disruptions (18).

The findings revealed that behavioral symptoms such as restlessness and reduced interest in routine activities were frequently reported among female university students, corroborating earlier study (17, 21), who highlighted that PMS-related behavioral changes significantly impair students’ daily routines and academic responsibilities. Similarly, a study observed that PMS symptoms including psychological and behavioral distress negatively influenced class attendance, concentration, and social interactions among Saudi university students. This is consistent with findings reported from other countries (2, 4, 6, 9, 20), where behavioral manifestations such as irritability, social withdrawal, and difficulty concentrating were shown to interfere with students’ educational performance and quality of life. A study in Riyadh also reported that more than half of the students experienced functional impairment, particularly in academic and household roles, due to PMS-related psycho-behavioral symptoms (9). These cumulative findings suggest that behavioral disruptions associated with PMS are not only common but also have substantial implications for student well-being, requiring integrated support within educational institutions.

Regression analyses further revealed that marital status, menstrual flow, and cycle length were significant predictors of physiological symptoms. Psychological symptoms were influenced by flow type, while behavioral symptoms were predicted by marital status, and flow type BMI was significantly associated with all three domains of PMS. Overweight and obese participants reported lower symptom severity compared to underweight peers. Although this may appear counterintuitive, similar patterns have been observed in prior research indicating that underweight individuals may have more hormonal imbalances or lower fat reserves, which can affect estrogen production and regulation (16–18). The finding aligns with previous studies suggesting that leaner body types may be more vulnerable to emotional instability during the premenstrual phase (17). Menstrual flow intensity, age at menarche, and academic background were also significantly associated with PMS severity. Heavy menstrual flow was strongly linked to higher symptom burden across all domains, likely due to hormonal and prostaglandin dysregulation (8, 12, 23, 26, 27). Early menarche (≤13 years) was associated with more behavioral symptoms, supporting evidence that early hormonal exposure may predispose adolescents to mood and behavioral changes (10, 28–31). Furthermore, students from non-healthcare departments exhibited significantly higher psychological and behavioral symptoms, possibly due to limited health literacy or coping mechanisms compared to their healthcare peers (3, 5, 10, 17, 28). These insights emphasize the need for targeted PMS screening, health education, and mental health interventions for specific high-risk student groups.

Strengths of this study include the use of a standardized and validated tool of Premenstrual Syndrome Scale (PMSS) to comprehensively assess the physiological, psychological, and behavioral dimensions of PMS. The relatively large sample size enhances the representativeness of findings among undergraduate female students in the region. The study also incorporated a broad range of demographic and menstrual characteristics, enabling detailed correlation and association analyses. By capturing symptom severity and its potential impact on academic performance, the study offers practical insights relevant for institutional policy development.

However, several limitations must be acknowledged. First, the use of a convenience sampling technique limits the generalizability of the findings, as the participants may not represent the broader population of female university students. Second, the cross-sectional design restricts the ability to infer causality between premenstrual symptoms and associated demographic or menstrual factors; longitudinal studies are needed to examine symptom progression over time. Additionally, data were self-reported, including BMI, menstrual characteristics, and symptom severity, which may introduce recall bias and social desirability bias. The reliance on subjective reporting may affect the accuracy of BMI classification and the intensity of symptoms reported. Finally, several important factors known to influence PMS such as psychological stress, dietary habits, and physical activity were not assessed in this survey. Notably, the study did not differentiate or include specific clinical menstrual disorders such as menorrhagia or menometrorrhagia, which are often associated with pronounced physiological and psychological distress. Future studies should incorporate these conditions to comprehensively examine their relationship with behavioral and emotional symptoms of PMS.

### 4.1 Study implications

The findings of this study highlight critical implications for both academic institutions and public health policymakers, particularly in the Saudi Arabian higher education context. The significant associations between premenstrual symptoms (physiological, psychological, and behavioral) and variables such as BMI, early menarche, and menstrual flow suggest that tailored health strategies are urgently needed. Educational institutions should prioritize integrating premenstrual syndrome (PMS) awareness into student health education and ensure the availability of trained health clinic staff to provide support. Additionally, academic policies must be adapted to offer flexibility and accommodations for students experiencing severe PMS, which can impact academic performance and well-being. These measures will not only improve students’ health outcomes but also enhance their academic engagement and retention. The study underscores the necessity for holistic, culturally sensitive interventions that address both physical and psychological aspects of PMS among female university students.

## 5 Conclusion

This study found that menstrual flow severity, BMI, and academic department are significant correlates of PMS symptoms in Saudi female students. Interventions should focus on early screening, health literacy campaigns, and campus support structures. This study highlights the high prevalence of premenstrual syndrome among female university students, with the majority experiencing moderate to higher physiological, psychological, and behavioral symptoms. Age and body weight were significantly associated with symptom severity, emphasizing the need for targeted interventions. While most symptoms were not severe, their cumulative impact on daily functioning and academic performance is notable. Given the burden of PMS among university students, especially within the Saudi context, proactive measures are warranted. Educational institutions should integrate PMS awareness, early screening, and supportive services into student health systems. Nursing educators and administrators are encouraged to adopt tailored health education initiatives, provide accessible counseling, and implement flexible academic accommodations. These efforts can collectively enhance students’ physical well-being, psychological resilience, and academic success.

## Data Availability

The raw data supporting the conclusions of this article will be made available by the authors, without undue reservation.
